# Case Report Series: Genetic and clinical characterization of long QT syndrome in admixed Ecuadorian patients and its implications for sudden cardiac death risk

**DOI:** 10.3389/fcvm.2026.1680300

**Published:** 2026-02-12

**Authors:** Elius Paz-Cruz, Viviana A. Ruiz-Pozo, Santiago Cadena-Ullauri, Patricia Guevara-Ramírez, Rafael Tamayo-Trujillo, Rita Ibarra-Castillo, José Luis Laso-Bayas, Leonel Meza-Chico, Ana Karina Zambrano

**Affiliations:** 1 Universidad UTE, Facultad de Ciencias de la Salud Eugenio Espejo, Centro de Investigación Genética y Genómica, Quito, Ecuador; 2 Clinical Cardiac Electrophysiologist, Quito, Ecuador

**Keywords:** cardiovascular disease, Ecuadorian, genetics, genomics, healthcare

## Abstract

Long QT syndrome (LQTS) is a hereditary cardiac channelopathy associated with delayed ventricular repolarization and increased risk of life-threatening arrhythmias and sudden cardiac death. We report three Ecuadorian patients with LQTS, each presenting distinct clinical features and carrying pathogenic or likely pathogenic variants in KCNH2 or KCNQ1. Subject A, an 18-year-old woman with exertion-related syncope and a QTc of 520 ms, was diagnosed with LQT2 due to a KCNH2 p.Ala614Val variant. Subject B, a 3-year-old girl with congenital deafness and a QTc of 580 ms, was diagnosed with Jervell and Lange-Nielsen syndrome (JLNS), harboring a homozygous KCNQ1 p.Arg192Cys variant. Subject C, a 44-year-old man with recurrent syncope misdiagnosed as epilepsy and a strong family history of sudden death, was found to carry a KCNH2 p.Val612Met variant and had a QTc of 600 ms. All variants were classified according to ACMG/AMP guidelines and supported by in silico and functional data. Ancestry analysis provided additional genomic context in this admixed population. These cases underscore the clinical utility of integrating ECG findings, genetic testing, and ancestry-informed interpretation to improve diagnostic accuracy and personalize management in patients with inherited arrhythmia syndromes.

## Introduction

Long QT syndrome (LQTS) is a potentially life-threatening arrhythmogenic disorder characterized by a prolonged QT interval on electrocardiogram (ECG), abnormal T-wave morphology, and an increased risk of arrhythmias, syncope or cardiac arrest, which may lead to sudden cardiac death (SCD), especially in young individuals (1–3). The condition is primarily inherited in an autosomal dominant manner, and approximately 80% of cases have a confirmed genetic etiology (4).

The estimated global prevalence of LQTS ranges from 1 in 2,000 to 1 in 2,500 individuals, with cases reported across all ethnic groups (2, 3). However, due to variable expressivity and incomplete penetrance, the true prevalence may be underestimated (5). To date, no epidemiological data are available regarding the prevalence or incidence of LQTS in the Ecuadorian population, and genomic information is even more scarce.

LQTS is intrinsically linked to an elevated risk of SCD due to its underlying electrophysiological substrate (6). The defining feature, prolonged ventricular repolarization, creates a proarrhythmic myocardial milieu that predisposes affected individuals to malignant ventricular arrhythmias (7, 8). Among these, torsades de pointes (TdP), a hallmark polymorphic ventricular tachycardia of LQTS, frequently manifests as syncope and may rapidly degenerate into ventricular fibrillation, culminating in cardiac arrest and death if not promptly addressed (7–9). The principal precipitant of TdP is QT interval prolongation; thus, the corrected QT interval (QTc) on electrocardiography serves as a pivotal prognostic marker, with greater prolongation correlating with heightened arrhythmic risk (10).

Furthermore, multiple factors contribute to the risk of SCD in LQTS, including inherited mutations in ion channel genes (e.g., KCNQ1, KCNH2, SCN5A), family history of sudden death, drug-induced QT prolongation, electrolyte imbalances (notably hypokalemia and hypomagnesemia), and environmental or physiological triggers such as intense physical exertion or emotional stress (10–12).

LQTS has been genetically classified into 17 subtypes, involving mutations in at least 11 known genes that encode ion channel components or associated regulatory proteins (13). These mutations primarily affect potassium, sodium, or calcium channels, leading to abnormal cardiac repolarization and increased arrhythmogenic risk (2, 14, 15). The three most common subtypes, include LQT1, LQT2, and LQT3, which account for the majority of genetically confirmed cases and exhibit distinct genotype–phenotype correlations (14). For example, LQT2 is caused by mutations in the KCNH2 gene, which encodes the *α*-subunit of the rapidly activating delayed rectifier potassium channel (IKr) (2, 16). This channel plays a critical role in ventricular repolarization and is predominantly expressed in cardiac and neuronal tissues (15).

Clinical management of LQTS is guided by subtype, symptom burden, and risk stratification (17, 18). Beta-blockers remain the cornerstone of therapy, particularly in LQT1 and LQT2, where they reduce adrenergic stimulation and arrhythmic events (13). In high-risk individuals, such as those with recurrent syncope, markedly prolonged QTc, or a family history of SCD, implantable cardioverter-defibrillators (ICDs) may be indicated. However, identifying candidates for ICD therapy, especially among pediatric and asymptomatic carriers, remains a clinical challenge (17, 18).

The primary aim of this study is to describe the genetic and clinical characteristics of three Ecuadorian patients with LQTS, a condition for which only isolated, genetically confirmed case reports have been published in Ecuador. This work integrates the three cases into a cohesive series, allowing a comparative analysis of clinical variability and genotype–phenotype correlations within an admixed population. Furthermore, by applying ACMG/AMP variant classification guidelines and incorporating ancestry-informative marker analysis, the study provides a comprehensive perspective on the molecular spectrum of LQTS. This approach highlights the underdiagnosis and clinical relevance of the condition in Ecuador and contributes to a broader understanding of inherited arrhythmia syndromes in Latin American populations.

## Case presentation

### Subject A

A 14-year-old woman was diagnosed with congenital LQTS, following electrocardiographic findings consistent with QT interval prolongation. She had experienced two syncopal episodes, one following physical exertion and another upon rising after a period of rest, which prompted a comprehensive clinical and electrocardiographic reassessment.

Electrocardiography demonstrated sinus rhythm at 62 beats per minute. The PR and QRS intervals were within normal limits, and there was no evidence of atrial or ventricular hypertrophy. The corrected QT interval (QTc), calculated using the Bazett, Fridericia, and Framingham formulas, was consistently prolonged, with a value of 0.52 s, exceeding the upper reference limit for her age and sex (Supplementary Figure S1).

Initial management included beta-blocker therapy. However, due to recurrent syncopal episodes and an elevated risk of malignant ventricular arrhythmias, an implantable cardioverter-defibrillator (ICD) was placed as a preventive measure against sudden cardiac death.

**Figure 1:**
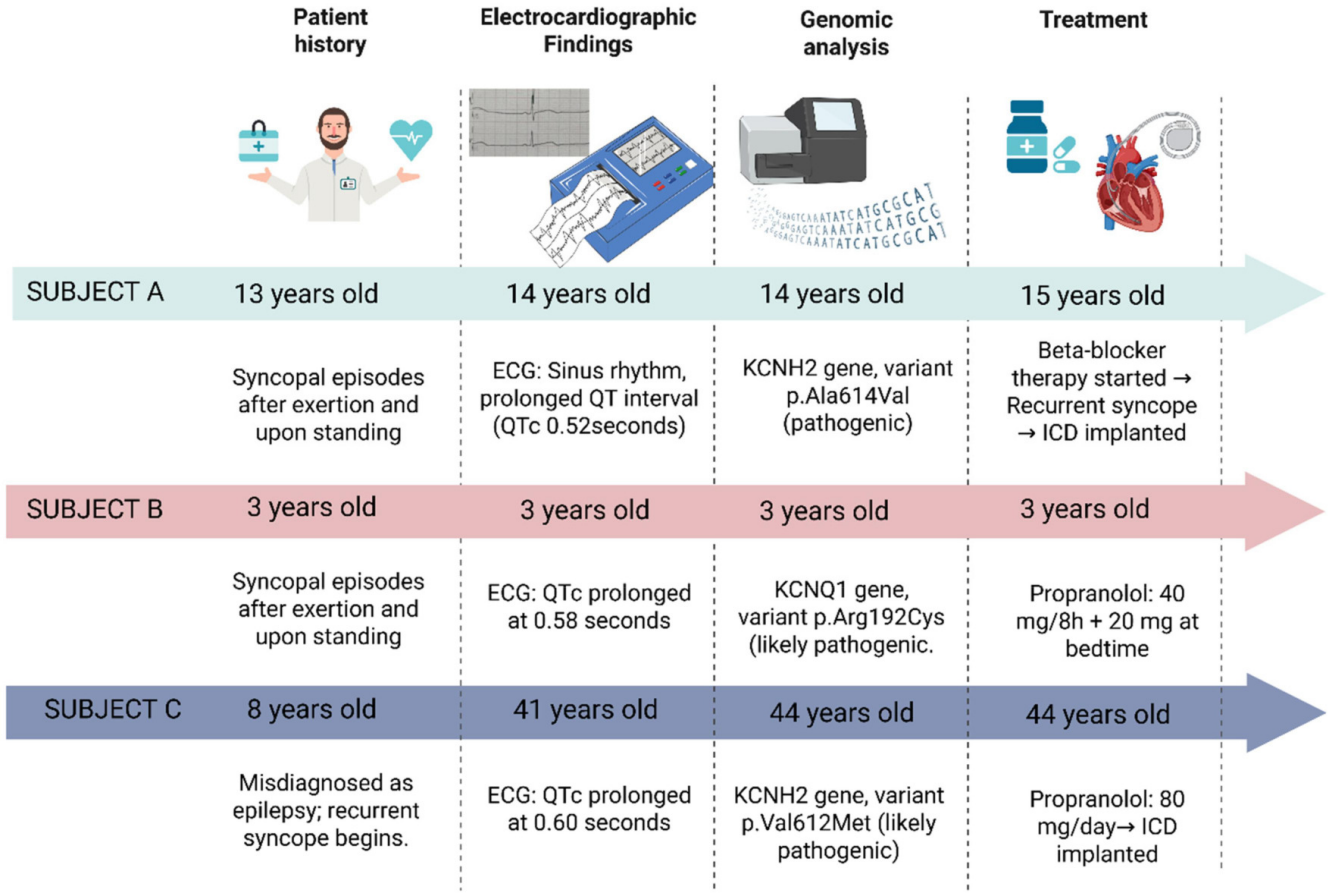
Schematic representation of the clinical history, diagnostic, and treatment in three Ecuadorian patients with genetically congenital long QT syndrome. Created with BioRender (https://www.biorender.com/).

As part of the diagnostic follow-up, genetic testing was performed to identify pathogenic variants associated with inherited cardiac channelopathies. A heterozygous pathogenic variant in the KCNH2 gene (Ala614Val) was identified (Table 1), confirming a definitive diagnosis of type 2 long QT syndrome (LQT2) (Figure 1).

**Table 1:** Clinical features, genetic findings, and ACMG/AMP classification criteria for subject A, B and C diagnosed with congenital long QT syndrome.

Case	Age	Gender	Clinical features	Gene	HGVSP DNA/HGVS protein reference	Consequence	Predicted effect	Genotype	ACMG-AMP codes applied^a^	Ancestry analysis^b^
Subject A	18	Woman	Two syncopal episodes (after exertion and upon standing).	*KCNH2*	NM_000238.3 c.1841C>T p.(Ala614Val)	Missense	Pathogenic	Heterozygous	PP1, PS3, PS2, PM1, PP2, PM2, PP3.	AF: 8.3% EU: 40.3%| NA: 51.3%
Subject B	3	Woman	Severe bilateral sensorineural hearing loss. QTc prolongation suggestive of ventricular repolarization abnormality.	*KCNQ1*	NM_000218.2 c.574C>T p(Arg192Cys)	Missense	Likely Pathogenic	Homozygous	PM1, PP2, PM2, PP3, PM5.	AF: 9.6% EU: 43.5% NA: 46.9%
Subject C	44	Man	Recurrent syncope since age 8. Diagnosed with Long QT syndrome in adulthood.	*KCNH2*	NM_000238.3 c.1834G>A p(Val612Met)	Missense	Likely Pathogenic	Heterozygous	PM1, PP2, PM2, PM5, PP3.	AF: 13.4% EU: 56.0% NA: 30.6%

a PP1: Co-segregation with disease in multiple affected family members, PS3: Well-established *in vitro* or *in vivo* functional studies supportive of a damaging effect on the gene or gene product, PS2: *De novo* (both maternity and paternity confirmed) in a patient with the disease and no family history, PM1: Located in a mutational hot spot and/or critical and well-established functional domain, PP2: Missense variant in a gene that has a low rate of benign missense variation and where missense variants are a common mechanism of disease, PM2: Absent from controls (or at extremely low frequency if recessive) in Exome Sequencing Project, 1000 Genomes or ExAC, PP3: Multiple lines of computational evidence support a deleterious effect on the gene or gene product, PM5: Novel missense change at an amino acid residue where a different missense change determined to be pathogenic has been seen before.

b AF, African; EU, European; and NA, Native American.

### Subject B

A 3-year-old girl with profound bilateral sensorineural hearing loss underwent cochlear implantation. She had no prior history of syncope, seizures, or documented arrhythmias. However, a routine electrocardiogram revealed broad, deeply inverted T waves in leads V3 and V4, suggestive of abnormal ventricular repolarization.

The QTc was markedly prolonged at 0.58 s, a parameter well above the upper reference limit for pediatric populations (Supplementary Figure S1). In the context of congenital deafness and a QTc exceeding 0.45 s, a clinical diagnosis of Jervell and Lange-Nielsen syndrome (JLNS) was strongly suspected. Thus, the patient was initiated on oral propranolol at 40 mg every 8 h, with an additional 20 mg at bedtime.

As part of the diagnostic follow-up, genetic testing identified a likely pathogenic variant in the KCNQ1 gene (Arg192Cys) (Table 1), supporting the diagnosis of JLNS. No additional variants were detected in other major LQTS–associated genes (Figure 1).

### Subject C

A 41-year-old man presented with a longstanding history of recurrent syncope. He had been diagnosed with epilepsy at the age of 8; however, no seizure activity was documented during subsequent evaluations. His family history was significant for multiple cases of sudden cardiac death on the maternal side: his daughter, maternal aunt, and the latter's granddaughter died suddenly at the ages of 12, 40, and 22 years, respectively. Additionally, two maternal cousins had received implantable cardioverter-defibrillators (ICDs) for arrhythmia management.

Electrocardiography revealed sinus rhythm with bradycardia at 56 beats per minute. Prominent U waves were observed, suggestive of delayed repolarization of the Purkinje fibers. The PR and QRS intervals were within normal limits, and there was no electrocardiographic evidence of atrial or ventricular enlargement. The QTc was consistently prolonged, with a maximum value of 0.60 s, which substantially exceeds the upper reference threshold (Supplementary Figure S1).

Based on these findings, at 44-years old, a presumptive diagnosis of congenital LQTS was made. The patient was initiated on oral propranolol at a total daily dose of 80 mg. Subsequent genetic testing identified a likely pathogenic variant in the KCNH2 gene: c.1834G>A (p.Val612Met) (Table 1), thereby confirming a definitive diagnosis of type 2 long QT syndrome (LQT2) (Figure 1).

In summary, the three subjects carried missense variants in either *KCNH2* or *KCNQ1*, classified as pathogenic or likely pathogenic according to ACMG/AMP criteria. Two patients (Subjects A and C) harbored heterozygous variants in *KCNH2*, supporting a diagnosis of type 2 long QT syndrome (LQT2). Subject B presented a homozygous *KCNQ1* variant, consistent with the diagnosis of JLNS. All identified variants were rare or absent in global population databases, suggesting a pathogenic potential. The applied ACMG/AMP evidence codes provided supporting evidence for potential causative role of these variants (Table 1). No additional variants classified as likely pathogenic or pathogenic were identified in other genes included in the TruSight Cardio panel.

Furthermore, Ancestry-informative marker analysis showed that all subjects exhibited an admixed genomic ancestry. European ancestry ranged from 40.3% to 56.0%, Native American from 30.6% to 51.3%, and African from 8.3% to 13.4% (Table 1). These data reveal notable inter-individual variability in ancestral composition and could potentially influence the distribution and phenotypic expression of rare cardiovascular variants.

## Methodology

### Sample collection and DNA extraction

Peripheral blood samples were collected in EDTA tubes. Genomic DNA was extracted using the PureLink™ Genomic DNA Mini Kit (Invitrogen), following the manufacturer's protocol. DNA concentration and purity were assessed using spectrophotometric and fluorometric methods. DNA integrity was evaluated by agarose gel electrophoresis.

### Next-generation sequencing (NGS)

Genomic analysis was conducted at the Centro de Investigación de Genética y Genómica (CIGG), Universidad UTE. Targeted sequencing was performed using the TruSight Cardio panel (Illumina), which includes 174 genes associated with 17 inherited cardiovascular conditions. Libraries were prepared and sequenced on the MiSeq platform, according to the manufacturer's instructions.

### Bioinformatic analysis and variant interpretation

Sequencing data were processed using the DRAGEN Enrichment pipeline (v3.9.5). The analysis workflow included: 1) alignment to the reference genome (hg38), 2) identification of genetic variants from the sequencing reads, 3) annotation of those variants with relevant information, and 4) filtering and prioritization of variants likely to cause the patient's condition.

Variant interpretation was performed using two complementary platforms: Variant Interpreter (Illumina) and Franklin by Genoox. Both tools were used to enhance the robustness of variant classification. Notably, Franklin applies the American College of Medical Genetics and Genomics/Association for Molecular Pathology (ACMG/AMP) guidelines for standardized variant interpretation. Variants of uncertain significance (VUS) were evaluated but not reported in detail, as they lacked sufficient clinical or functional evidence. Furthermore, copy number variants (CNV) analysis did not identify clinically relevant deletions or duplications.

### Ancestry analysis

Ancestry inference was performed using a multiplex PCR assay targeting 46 ancestry-informative insertion/deletion markers (AIM-InDels), following the protocol described by Zambrano et al. (2019). PCR products were separated and detected using a 3500 Genetic Analyzer (Applied Biosystems). Data acquisition was conducted with Data Collection Software v3.3, and genotyping analysis was performed using GeneMapper Software v5.0 (Applied Biosystems).

## Discussion

This case series contributes to the existing LQTS literature by providing an ancestry-informed clinical and genomic characterization of congenital LQTS in Ecuadorian patients, an admixed population that remains markedly underrepresented in inherited arrhythmia studies (19). The manuscript focuses on integrating electrocardiographic evaluation, NGS-based molecular diagnosis, ACMG/AMP variant interpretation, and ancestry-informative marker analysis, this work highlights the clinical heterogeneity of LQTS and the importance of precision medicine approaches in diverse populations.

Diagnosis of LQTS relies primarily on ECG findings, particularly QT interval prolongation and abnormal T-wave morphology; however, the identification of pathogenic variants, in genes known to cause LQTS, is sufficient for a diagnosis (4). To date, 17 genes have been associated with LQTS, most of which encode cardiac ion channels or their regulatory proteins, leading to disruptions in channel function and cardiac repolarization (4). Three main LQTS subtypes are distinguished based on the affected gene, with *KCNQ1* associated with LQTS type 1, *KCNH2* with type 2, and *SCN5A* with type 3 (4). Notably, clinical manifestations vary according to the specific genetic subtype. The present case report describes variants in the *KCNQ1* and *KCNH2* genes.

The gene *KCNQ1* encodes a pore-forming *α* subunit of a voltage-gated potassium (Kv) channel crucial for cardiomyocyte excitability and is also implicated in the normal development of the myocardium and inner ear, among other tissues (20–22). Pathogenic variants in this gene are strongly associated with LQTS type 1 and may also result in sensorineural hearing loss (21). *KCNQ1*-associated LQTS carries increased mortality in women older than 13 years, with a cumulative probability of cardiac events reaching 44% by age 50, and a 5% risk of SCD. These cardiac events may be triggered by exercise (4).

Furthermore, pathogenic variants in this gene have been associated with JLNS, a condition characterized by congenital sensorineural hearing loss, a prolong QTc interval (greater than 0.45 s), and an increased risk of sudden cardiac death (23). Accurate diagnosis of JLNS is crucial, as approximately 50% of affected individuals experience cardiac events before the age of three, and without treatment, over half die before reaching the age of 15 due to a greater than 25% risk of sudden cardiac death (23–25). JLNS follows an autosomal recessive inheritance pattern, requiring affected individuals to be homozygous for pathogenic variants in the gene *KCNQ1* (23). Notably, Subject B presents with all the aforementioned clinical features of JLNS, and genetic analysis revealed that she is homozygous for a likely pathogenic variant in the *KCNQ1* gene, further supporting the diagnosis of JLNS.

In this study, the subject B carries a variant classified as Likely pathogenic per American College of Medical Genetics and Genomics (ACMG) guidelines (26). This classification is based on evidence from functional studies indicating the variant lies in a mutational hotspot or a critical functional domain; additionally, *KCNQ1* is known to have a low rate of benign missense variation and a high susceptibility to pathogenic missense changes (27, 28). Furthermore, the variant is extremely rare in population databases (29), and multiple *in-silico* prediction tools suggest deleterious effects on protein function. Importantly, different amino acid substitutions at the same residue have been associated with pathogenicity (27, 28, 30). For instance, Vanoye et al., (2019) showed that the Arg192Cys variant altered ion channel currents, suggesting a functional impact on cellular physiology (31). Similarly, Suktitipat et al., (2017) identified this variant in a patient who suffered sudden unexpected death syndrome in Thailand; however, computational predictors classified the variant as tolerable, indicating that further research is needed to clarify its pathogenic significance (32).

The gene *KCNH2* encodes the α subunit of a voltage-gated inwardly rectifying potassium channel, expressed in multiple tissues including cardiac muscle and neural cells. Pathogenic variants in this gene have been linked to LQTS type 2 (20, 22). Notably, *KCNH2*-associated LQTS is associated with higher mortality in males aged 5–15 years, with a cumulative probability of cardiac events reaching 49% by age 50, and a 3% risk of SCD (4). Furthermore, the lifelong cumulative risk of cardiac events is higher in females (33). These cardiac events can be triggered by auditory stimuli, sleep, exercise, or emotional stress (4).

Two patients in this report (subjects A and C) carry variants in *KCNH2*. The subject A harbors the Ala614Val variant, classified as Pathogenic according to ACMG criteria. This classification was based on segregation data, functional and *de novo* data, population frequency, and *in-silico* predictions indicating a deleterious effect on the gene or protein product. The variant is extremely rare in the gnomAD database, and it has been shown to cosegregate with LQTS phenotypes in families (27–29, 34–36). Sakaguchi et al., (2008) reported a case of LQTS triggered by an H(1)-receptor antagonist in a patient carrying the same variant, suggesting that drug–gene interactions can exacerbate disease severity (37). Further research has shown that this genetic variant is especially important to consider when administering drugs as it has been demonstrated that carriers of the variant have an increased to susceptibility to worsening of the symptoms due to pharmacogenetic interactions (36, 38, 39).

Similarly, the subject C harbors a variant categorized as Likely Pathogenic per ACMG guidelines. The classification was based on functional data indicating the variant resides in a mutational hotspot or critical functional domain, and on the established pathogenicity of missense changes in *KCNH2* (27, 28). The variant is rare in population databases (29), and multiple computational tools predict deleterious effects on protein function. Additionally, different amino acid substitutions at the same site have been described as pathogenic (27, 28). Although current evidence suggests potential pathogenicity, further studies are needed to fully elucidate the clinical impact of this variant.

All reported variants were classified as pathogenic or likely pathogenic according to ACMG/AMP criteria, providing a robust molecular basis for diagnosis. VUS were evaluated but not reported due to the absence of sufficient clinical evidence supporting its role in disease. CNV analysis was also performed as part of the bioinformatic pipeline and did not identify clinically relevant deletions or duplications.

Moreover, the strong association between QTc interval duration and the risk of SCD has been well established (33, 34). Studies across multiple populations have shown that longer QTc intervals are correlated with a significantly increased risk of SCD (34). Notably, researchers have reported a 0.7% increase in SCD risk for each additional millisecond of QTc prolongation (33). This elevated risk is particularly pronounced in young adults, with a median age of 32 years among individuals who have experienced SCD associated with prolonged QT intervals (35).

An important and novel aspect of this study is the incorporation of ancestry-informative marker analysis to contextualize genetic findings within Ecuador's admixed population., given that genetic ancestry can significantly influence human well-being, including cardiovascular health (40). Certain CVDs can disproportionately affect specific ethnic groups (41). For example, studies have shown that individuals of South Asian, Black African, or Caribbean descent exhibit a higher CVD risk compared with other populations, a disparity partly driven by genetic factors and environmental components such as diet, alcohol consumption, and smoking (42). Similarly, the Multi-Ethnic Study of Atherosclerosis (MESA) found the highest all-cause mortality rates among Black participants (26.4%), followed by White (23.3%), Hispanic (19.8%), and Chinese participants (18.0%) (43).

In this context, the present article incorporates ancestry-informative marker analysis for all three patients, which contextualizes the findings within Ecuador's admixed population and provides insights into how genetic background may modulate disease expression. Notably, one of the patients (Subject C) exhibited a predominant European genetic component, which is particularly relevant given that certain populations show an increased predisposition to carry mutations in specific genes (44). This observation underscores the importance of integrating ancestry data into clinical genomics and adds a novel dimension to our work. To our knowledge, this is the first report in Latin America to present an ancestry-informed genetic interpretation of LQTS.

These findings collectively highlight the need for an integrative approach to healthcare that considers not only the clinical and molecular aspects of disease but also the ancestral genetic background, which can facilitate the development of personalized therapies, leading to improved diagnosis, treatment outcomes, and quality of life.

## Conclusions

This case series highlights the clinical and genetic heterogeneity of congenital LQTS in Ecuadorian patients, highlighting the importance of generating population-specific data in an admixed population that remains underrepresented in research. The application of NGS enabled molecular diagnoses, directly impacting clinical management. Furthermore, integrating genetic findings with electrocardiographic evaluation supports risk stratification and disease management, including personalized pharmacologic treatment and device implantation. Collectively, these findings emphasize the value of precision medicine approaches to improve diagnosis, prevention of sudden cardiac death, and long-term outcomes in Ecuadorian and other admixed populations.

## Data Availability

The datasets generated and/or analyzed during the current study are available in the Sequence Read Archive (SRA) repository, BioProject number PRJNA1418113. For more information, please contact the corresponding author AZ (anazambrano17@hotmail.com).

## Supplementary material

Supplementary Figure S1 Representative electrocardiographic tracings from three Ecuadorian patients with congenital Long QT Syndrome. ECG recordings demonstrate prolonged ventricular repolarization with characteristic abnormalities, including QT interval prolongation, marked T-wave inversion, and prominent U waves, particularly in precordial leads. The electrocardiographic patterns are consistent with genetically confirmed Long QT Syndrome associated with pathogenic or likely pathogenic variants in *KCNH2* and *KCNQ1*.
