# Supplementary figures and images for: Case Report Series: Genetic and clinical characterization of long QT syndrome in admixed Ecuadorian patients and its implications for sudden cardiac death risk
Source: Front Cardiovasc Med. 2026 Feb 12;13:1680300. doi: 10.3389/fcvm.2026.1680300 (PMC12936859; doi:10.3389/fcvm.2026.1680300)

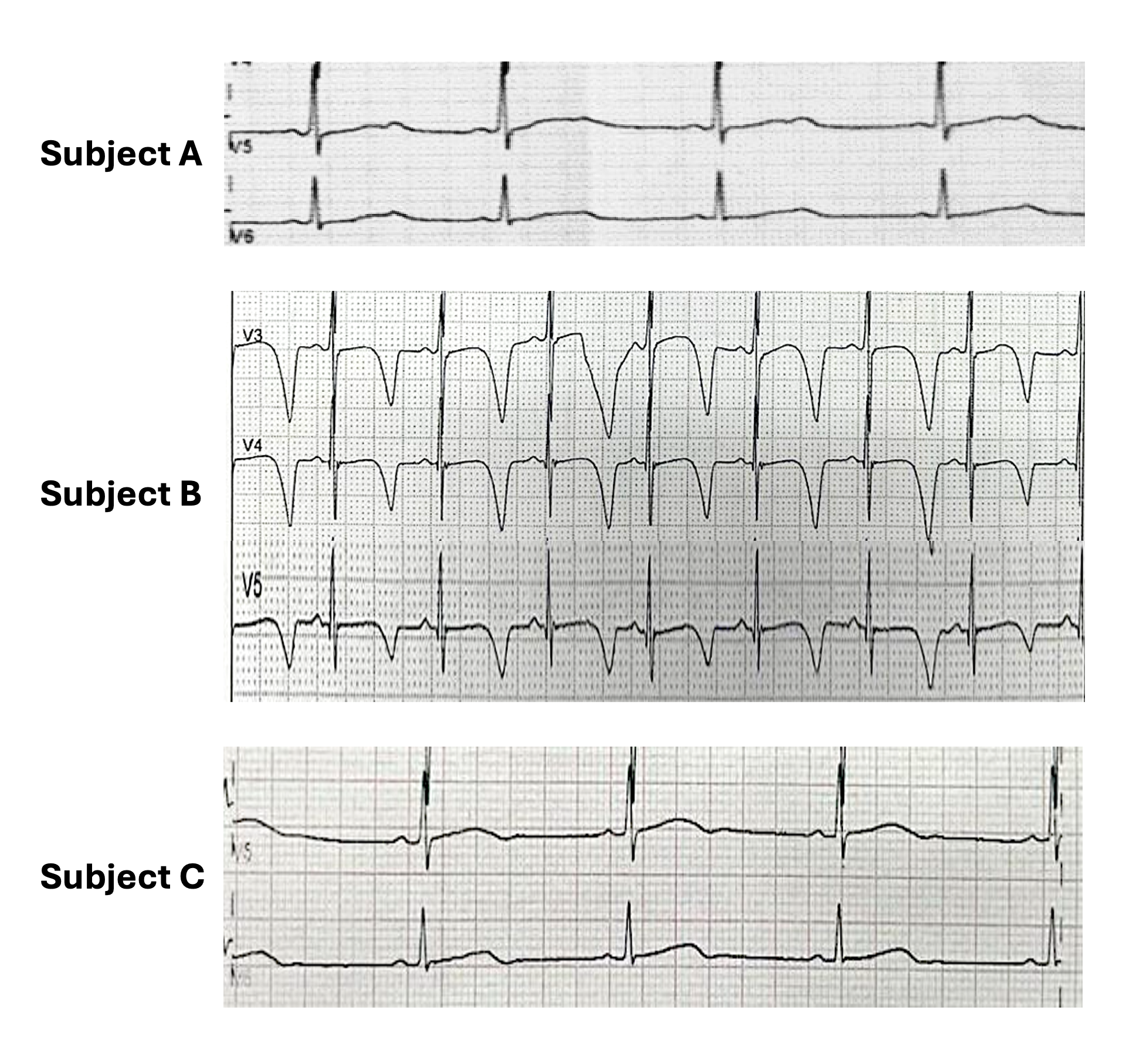

Supplement: Supplementary Figure S1 — Representative electrocardiographic tracings from three Ecuadorian patients with congenital Long QT Syndrome. ECG recordings demonstrate prolonged ventricular repolarization with characteristic abnormalities, including QT interval prolongation, marked T-wave inversion, and prominent U waves, particularly in precordial leads. The electrocardiographic patterns are consistent with genetically confirmed Long QT Syndrome associated with pathogenic or likely pathogenic variants in KCNH2 and KCNQ1. [file Image1.tiff]
